# Transactions of the Gynecological Society of Chicago

**Published:** 1891-10

**Authors:** 


### TRANSACTIONS OF THE GYNECOLOGICAL SOCIETY OF CHICAGO.

REGULAR MEETING, MAY 22D, 1891.

The President, Dr. W. W. JAGGARD, in the Chair.

DR. CHRISTIAN FENGER* read a paper on "Ovariotomy during Pregnancy."

DR. KARL SANDBERG: I am very glad that Dr. Fenger has brought this subject up to-night, because it is one of great interest to all, and one that has not been discussed as fully as I think it ought to be. Dr. Fenger has covered the ground so thoroughly that there is not much to do except to indorse all he has said. As soon as we agree, and I presume that we all do, that ovariotomy is indicated in general in cases of ovarian cyst, the next point is to decide whether pregnancy contitutes any bar against this operation. Statistics show that the mortality of ovariotomy complicated with pregnancy is so far from being larger than the general mortality of ovariotomy that it really seems to be lower. Spencer Wells, in 1883, reported eleven cases of ovariotomy, with only one death. This would make less than ten per cent.

*See original article, page 458.

At that time, I think, Spencer Wells' general mortality was about twenty-five per cent. Lawson Tait reported about the same time eleven cases, also with one death, which would give about ten per cent. This is higher than his general mortality, but he attributes this one death to the use of the clamp, which was also used in the case of Spencer Wells' that died. Olshausen published last year a series of twenty-four cases of his own without a single death. Olshausen's mortality from ovariotomy in gen eral is about ten per cent. Martin has some few cases without a death. All this goes to show that there is no greater danger in performing an ovariotomy during pregnancy than outside of it.

Furthermore, it is to be considered whether ovariotomy is possible in all cases where pregnancy is the complication. It has been laid down as a rule by Olshausen, and I think by Hegar and Kaltenbach, that small tumors impacted in the pelvis that do not seem to be movable contraindicate ovariotomy. I am extremely glad that Dr. Fenger has reported a case to-night of exactly this kind, where ovariotomy was performed with success to mother and child; it shows that this difficulty is only imaginary, it is only lack of courage on the part of the operator. I believe that all these cases can be operated upon by laparotomy, and the tumor can be removed; and if we have any doubt about it we ought not to cause a premature birth or tap a doubtful cyst through the vagina, as long as we can make a small exploratory incision and ascertain just what the condition is. It appears from Dr. Fenger's report that even in those cases where the tumor is impacted in the pelvis under the .pregnant uterus, the uterus can be lifted up and turned out, the tumor removed, the pedicle secured, and the operation finished in good shape. But even if the tumor could not be lifted out, I do not see any great obstacle to tying the pedicle at the bottom of the pelvis with a long-handled, blunt needle, tying and cutting as you go along.

An objection has been made to this operation because of the number of cases resulting in abortion afterwards. This seems to be a little different with different operators. Spencer Wells, in his first list, had one case where he did not expect pregnancy and tapped a pregnant uterus. He made a Cæsarean section, removed all the contents, and sutured the uterus. That cannot be counted in, but of his other ten cases three aborted. In one he secured the pedicle with a clamp, and the patient aborted about ten hours afterwards, and died; he had another case that aborted six days, and one that aborted twenty-five days after the operation—that makes three out of ten. Lawson Tait reports eleven cases uniformly successful. Olshausen, on the other hand, seems to have had several abortions. In his first eight cases he reports two abortions. In his whole series of twenty-four he does not give the number, but says that some aborted right after the operation and some a few weeks or months after, and he ascribes it in all the cases to the operation. Hegar and Kaltenbach give the percentage as about forty, without giving the sources from which they draw this conclusion. I get the impression from this that the number of abortions resulting from this operation is, and will be, different with different operators. It was doubtless so that in Spencer Wells' case the use of the clamp caused immediate abortion, which we can understand on account of the traction on the pedicle. We will probably find in the future that some operators who have a large percentage of abortions in the performance of the operation adopt some procedure which is to blame for this. But even if we admit that there are a great number of abortions, the fact that in a great number of cases the operation can be performed without abortion occurring is sufficient to do away with this objection, because, in the first place, it has been proved by Olshausen that abortions do not increase the mortality, and, in the second place, if we do not do ovariotomy we will have to depend upon the production of premature labor as the principal means of getting over the obstacle.

In conclusion, I congratulate the society upon the fact of this excellent paper being read here, and I most heartily indorse the views of Dr. Fenger.

DR. A. REEVES JACKSON: My experience in the removal of ovarian cystic growths during coexistent pregnancy comprises two cases, which I will briefly relate. Some years ago a woman was placed under my care with enlargement of the abdomen of a doubtful character, and which was supposed by her physician to be due to hydramnios. She had been married twice. Her first marriage was childless, and after a widowhood of three years she married again. Menstruation had been regular up to six months prior to the time at which I saw her. Then it had suddenly ceased and she supposed herself pregnant. Prior to the cessation of menstruation, however, a tumor had appeared in the left side of the lower abdomen, and had attained about the size of a cocoanut. After menstruation ceased there was very rapid distention of the abdomen, and, inasmuch as the tumor could not be clearly distinguished, the enlargement was attributed to dropsy of the ovum. At the time of my examination the abdomen was enormously distended; the enlargement was symmetrical and seemed to occupy the entire abdominal cavity. The walls of the abdomen were thin, traversed by enlarged vessels, and the umbilicus was protruding.

From the history which was given of the rational and sympathetic symptoms of pregnancy which had occurred during the first three months, and the condition of the os uteri, which was easily within reach, I felt satisfied that the patient was pregnant and that she had also ovarian cyst together with some free abdominal fluid. Her condition at that time was an extremely critical one and the symptoms urgent. I was, however, reluctant to interfere in a radical way so long as it seemed that she might go on to the full term of utero-gestation. There were some unusually cogent reasons why she should bear a living child. But the symptoms in a few days became so much more alarming that I concluded to tap the cyst, which I did, removing by a small trocar some gallons of fluid having a soap-and-water appearance. She was relieved at once, and after the operation the uterus could be distinctly felt rising up to within an inch and a half of the umbilicus, and at the lower left side was distinguishable the basic portions of the tumor, consisting of numerous hard nodules, clearly corroborating the diagnosis of multilocular ovarian cyst.

In the course of six weeks the abdomen filled again, with even greater rapidity than before, and she was so emaciated and suffered so greatly that I felt it to be unsafe to longer defer operation. Accordingly I removed the cyst. The patient got on well in every way, but five days afterward labor set in, and she was delivered of a child developed to about eight months, which survived. She recovered after a rather tedious convalescence and has subsequently borne two children. In this case the pregnancy was known to be present, but in the second case on which I operated the pregnancy was not known—not even suspected. In June, 1888, the wife of a clergyman, 30 years of age, was brought to this city from Lincoln, Neb., suffering from a rapidly growing cyst which had commenced in the right side. It was clearly defined, and gave the impression of being a tumor which might weigh four or five pounds. The patient suffered severely from pain, which had commenced about nine months before. I learned subsequently that she had ceased menstruating two months before I saw her. I had no hesitation in at once removing the tumor. At the time of the operation the uterus was found somewhat enlarged and presenting the appearance of pregnancy, and I expressed the belief that the pregnancy was two and a half months advanced. The patient recovered, went home, and was safely delivered of a living child in the early part of the following December.

With regard to the propriety of removing an ovarian cyst during pregnancy, I hold that the decision should depend upon the circumstances of each case. It should not be done because pregnancy coexists; on the other hand, the operation should not be withheld because of accompanying pregnancy. Usually in a case in which interference becomes necessary, and a choice could be made, I should prefer to remove the cyst rather than to induce premature labor. I deem it the safer operation of the two. Nevertheless, when it may be safely done, I should defer ovariotomy until the fetus had attained viability. I cannot agree with the opinion expressed here that it is less dangerous to remove ovarian cyst during pregnancy than under other circumstances.

I believe, on the other hand, that any important abdominal operation is likely to induce premature labor or abortion, and hence, unless the symptoms were urgent, I should defer operation until some necessity arose. Some of the statistics which have been adduced here to-night are misleading, because they give the impression that ovariotomy is made safer by pregnancy. I cannot accept this. If a sufficient number of cases were known I feel confident that the results would not lead to this conclusion. In the case related by Dr. Fenger there could be no question about the propriety of removing the tumor. There was a cyst of dermoid character so situated as to be liable at any time to give rise to dangerous complications from the bursting of its walls. Such a result would be almost certain to occur during labor. But when the cyst is found occupying such a position as not to interfere with labor, it may be properly let alone until after parturition. I am very glad that this paper has been brought before us. The statistics gathered here are exceedingly valuable, and personally I thank Dr. Fenger for presenting the subject.

DR. A. H. FOSTER: I would state that during the temporary absence of the attendant I was called to a case of labor, in an old patient of mine who had lived with one husband for several years without having become pregnant. By reason of some incompatibility of body or mind the parties separated, and she had married a second time, and this was the fruit of the union. I found that one of our Fellows had done ovariotomy upon her when she was some two months advanced, but I do not know the particulars of the case. Our lamented Dr. Parks had treated her, knew about the case, and had followed it up. The labor, which was a forceps case, was very difficult. I have been called to the family only once since, and then in a great hurry this last winter, and found the child living and doing well.

DR. FRANKLIN H. MARTIN: I simply wish to put a case on record. The first laparotomy I performed was in 1883. The patient, aged 30, presented the following spmptoms: She had not menstruated for three months. During the time that menstruation should have occurred the pain was intense and there she had epileptic fits. The patient was examined, and a diagnosis of pregnancy made. About two and a half months afterwards a tumor was discovered to the right of the uterus about the size of a cocoanut. The discovery of this tumor so changed the aspect of affairs in my mind that pregnancy, while previously admitted, was really lost sight of, and I decided to perform a laparotomy. I made my preparations as liberally as preparations were in the habit of being made in those days, including steaming and thorough renovation of the house in which the patient was to be operated upon. I was assisted in the operation by Drs. McArthur, Bishop, Doering, and others. The abdomen was opened, and a tumor about the size of a cocoanut to the right of the ovary discovered, together with a confirmation of the pregnancy. The tumor was removed, the abdomen closed in the ordinary way, and the patient put to bed. She did fairly well until the fourth day, when she aborted with a fetus of about four months. This was my first laparotomy. I started in with the idea of doing one hundred laparotomies without a death. At the end of the sixth day after this operation my mortality in abdominal surgery was one hundred per cent., the patient dying of acute sepsis.

DR. CHRISTIAN FENGER, in closing the discussion, said: I thank the Society very much for their kind reception of my paper. There are, of course, a number of questions pertaining to this matter which I have been unable, on account of lack of literature, to go thoroughly into. For instance, the question, shall we make a distinction between tumors in the small pelvis and tumors in the abdomen; is there any choice of action when a tumor is located in the small pelvis; shall we wait and not do ovariotomy under certain circumstances? Tumors in the small pelvis, I think, indicate, as Schroeder says, immediate operation. These questions can only be solved by having observed a sufficient number of cases where both of these classes of tumors were left until the end of pregnancy, so as to see the real danger of each of them. I had hoped that the obstetricians, who have seen a large number of labors, might give us some of their experiences in this respect, and thus help to solve that part of the question.

